# Supplementary figures and images for: Failure to modulate reward prediction errors in declarative learning with theta (6 Hz) frequency transcranial alternating current stimulation
Source: PLoS One. 2020 Dec 3;15(12):e0237829. doi: 10.1371/journal.pone.0237829 (PMC7714179; doi:10.1371/journal.pone.0237829)

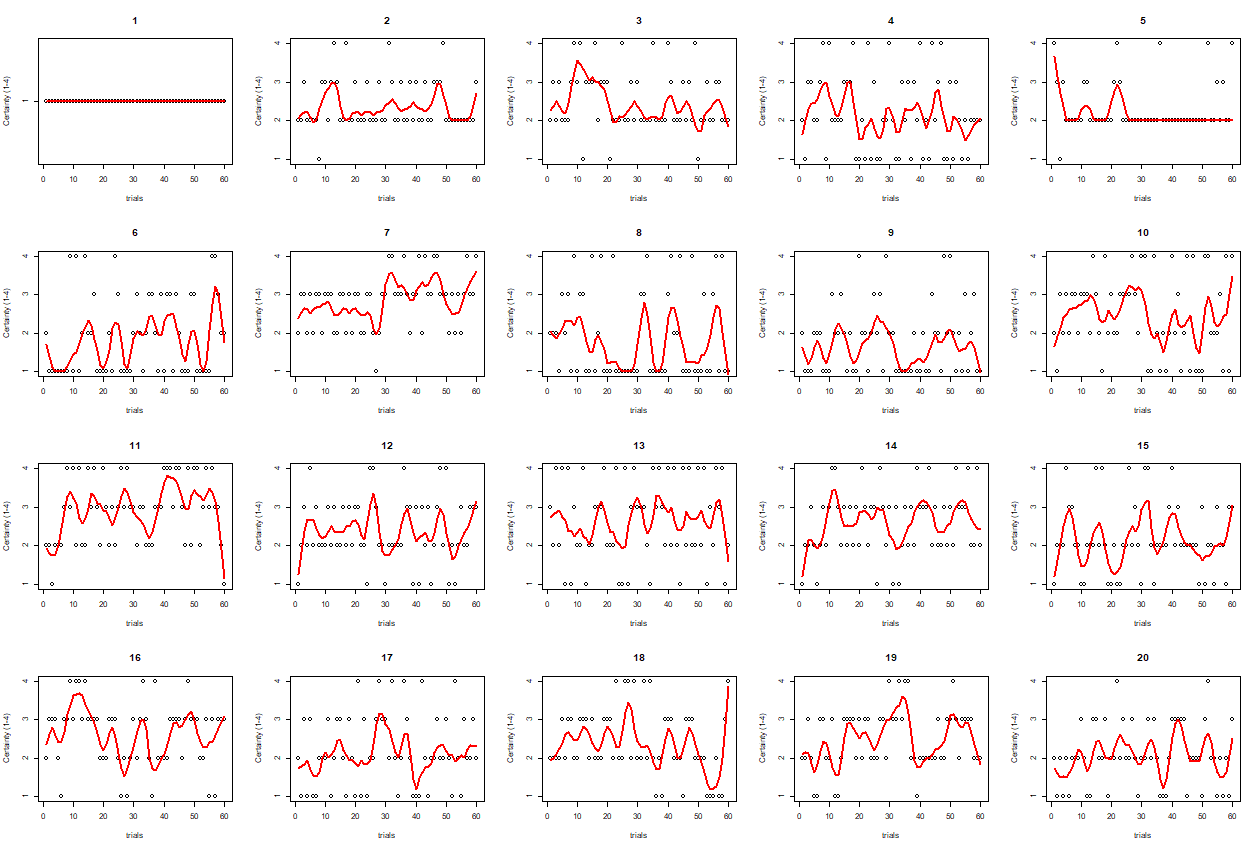

Supplement: S1 Fig — (TIFF) [file pone.0237829.s001.tiff]

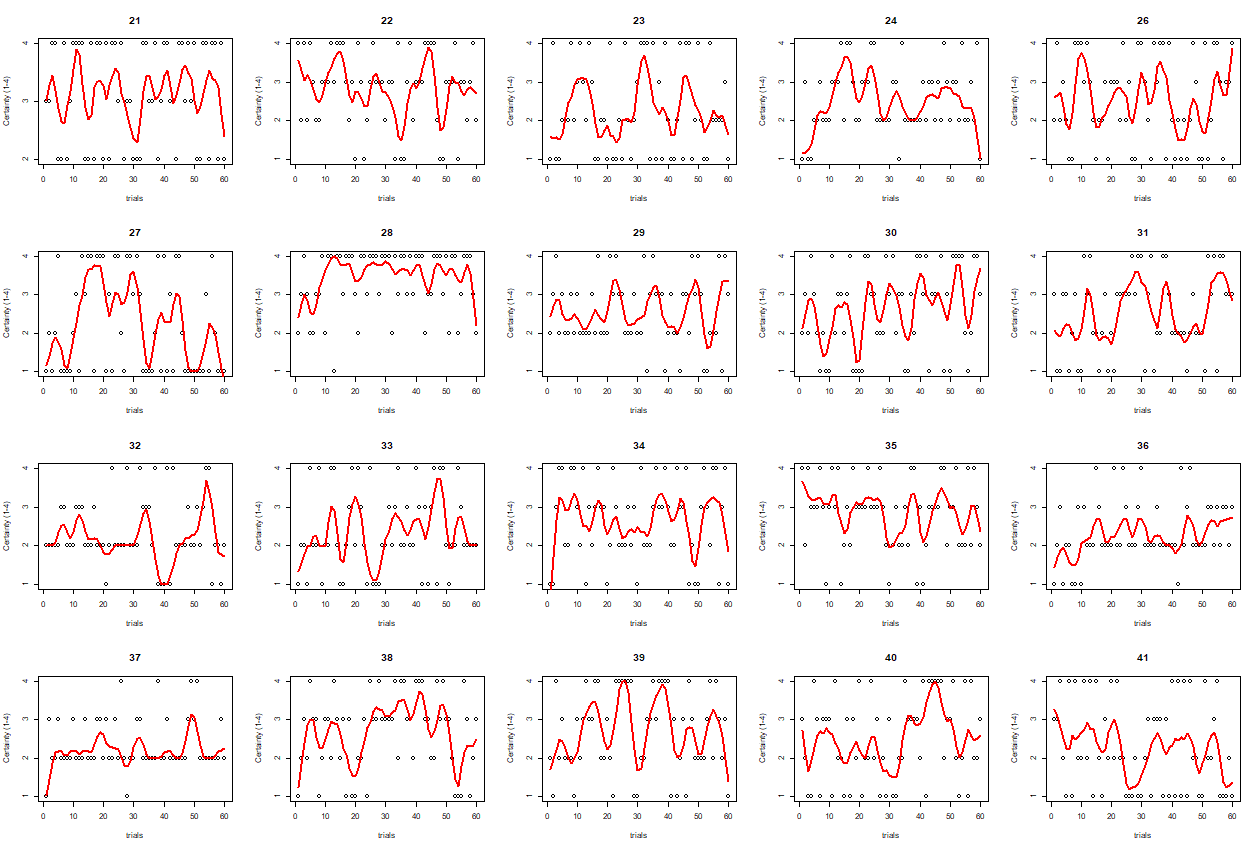

Supplement: S2 Fig — (TIFF) [file pone.0237829.s002.tiff]

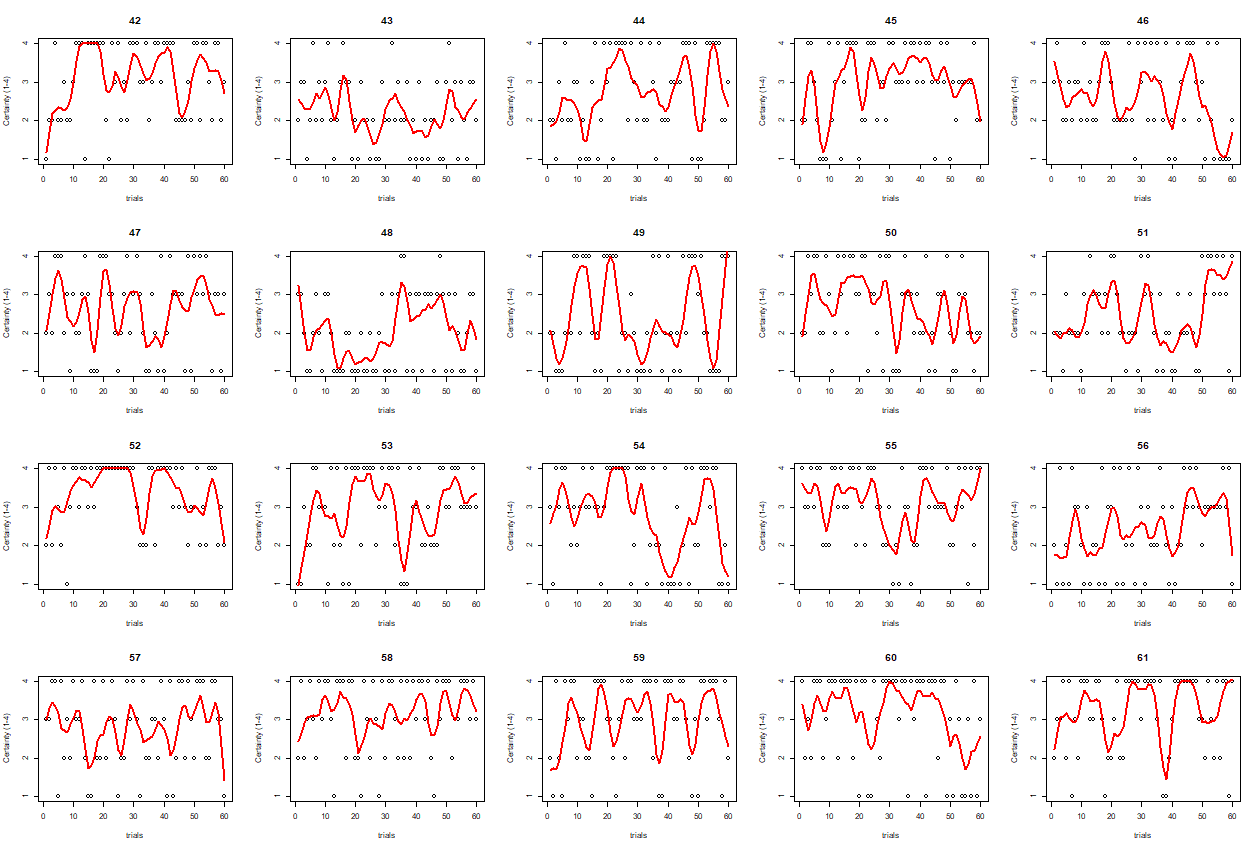

Supplement: S3 Fig — (TIFF) [file pone.0237829.s003.tiff]

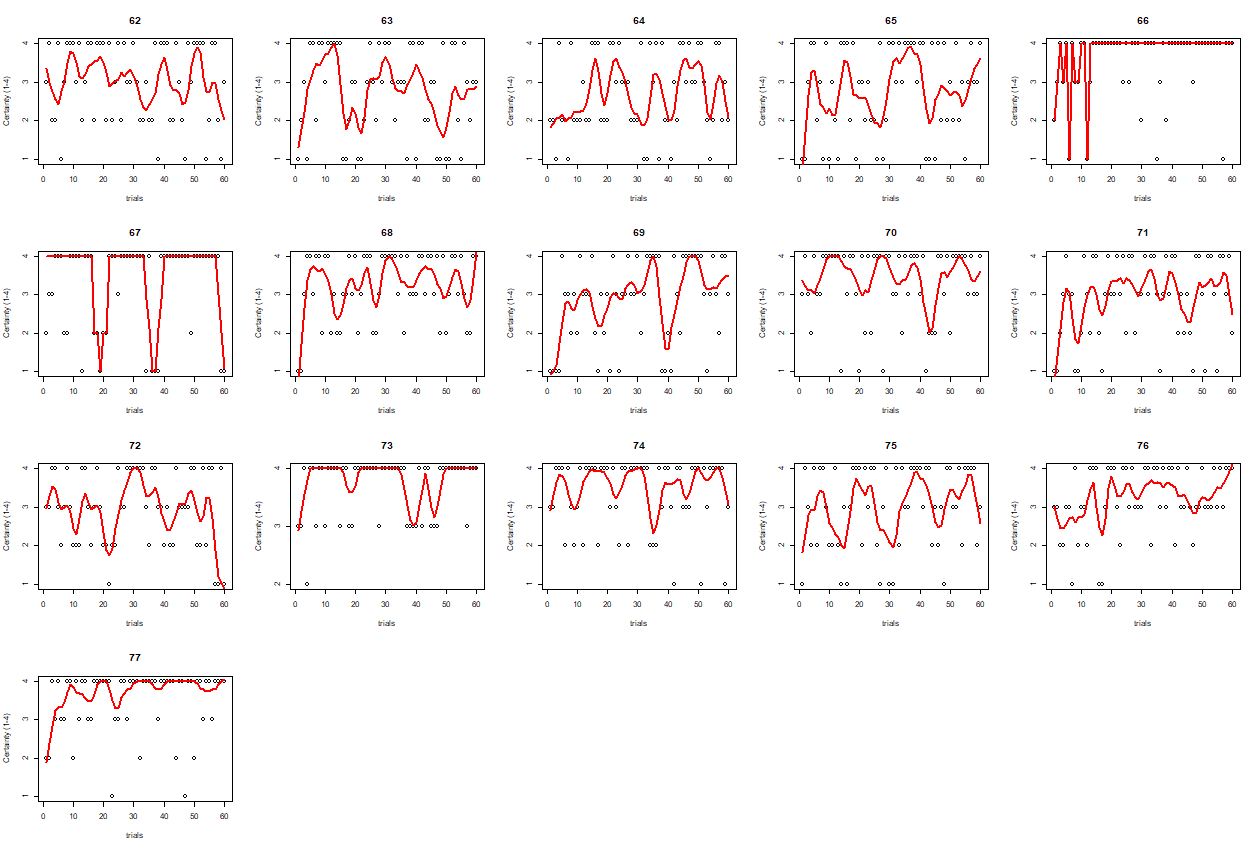

Supplement: S4 Fig — (TIFF) [file pone.0237829.s004.tiff]
