# Supplementary material for: Failure to modulate reward prediction errors in declarative learning with theta (6 Hz) frequency transcranial alternating current stimulation
Source: PLoS One. 2020 Dec 3;15(12):e0237829. doi: 10.1371/journal.pone.0237829 (PMC7714179; doi:10.1371/journal.pone.0237829)
Supplement: S1 Table — (DOCX) [file pone.0237829.s005.docx]

**S1 Table. Stimulus Material Practice Set: 6 Dutch Words.**

| zee | bier | hemel | koning | schaduw | handdoek |
| --- | --- | --- | --- | --- | --- |
