# Supplementary material for: Failure to modulate reward prediction errors in declarative learning with theta (6 Hz) frequency transcranial alternating current stimulation
Source: PLoS One. 2020 Dec 3;15(12):e0237829. doi: 10.1371/journal.pone.0237829 (PMC7714179; doi:10.1371/journal.pone.0237829)
Supplement: S2 Table — (DOCX) [file pone.0237829.s006.docx]

**S2 Table. Stimulus Material Practice Set: 24 Japanese Words.**

| ari | sen | ashi | yagi | ahiru | baggu |
| --- | --- | --- | --- | --- | --- |
| judan | kaeru | kutsu | nagai | tokei | zubon |
| beruto | idoboro | heishi | kujira | namida | sharin |
| shiten | tokage | chibusa | enpitsu | bengoshi | toriniku |
