# Supplementary material for: Failure to modulate reward prediction errors in declarative learning with theta (6 Hz) frequency transcranial alternating current stimulation
Source: PLoS One. 2020 Dec 3;15(12):e0237829. doi: 10.1371/journal.pone.0237829 (PMC7714179; doi:10.1371/journal.pone.0237829)
Supplement: S3 Table — (DOCX) [file pone.0237829.s007.docx]

**S3 Table. Stimulus Material: 60 Dutch Words.**

| bed | bes | kat | mug | pap | sok |
| --- | --- | --- | --- | --- | --- |
| vis | zon | boot | brug | doos | ezel |
| hond | kist | knie | lamp | mand | neus |
| raam | spin | tand | vlag | vork | wolf |
| zuil | agent | appel | beker | bloem | brief |
| brood | emmer | hamer | kaars | lepel | olijf |
| plank | schip | slang | steen | stift | stuur |
| trein | cadeau | heuvel | ladder | paling | parfum |
| schaar | sigaar | tomaat | vinger | winkel | gordijn |
| lichaam | plafond | sleutel | zakdoek | kampvuur | schouder |
