# Supplementary material for: Failure to modulate reward prediction errors in declarative learning with theta (6 Hz) frequency transcranial alternating current stimulation
Source: PLoS One. 2020 Dec 3;15(12):e0237829. doi: 10.1371/journal.pone.0237829 (PMC7714179; doi:10.1371/journal.pone.0237829)
Supplement: S4 Table — (DOCX) [file pone.0237829.s008.docx]

**S4 Table. Stimulus Material: 240 Swahili Words.**

| jua | adui | afya | aibu | basi | bega |
| --- | --- | --- | --- | --- | --- |
| buli | dari | duka | elfu | geza | goti |
| haki | hofu | jiwe | kazi | kioo | kisu |
| kiti | kito | leso | maji | mali | maua |
| mbwa | mkuu | moyo | mvua | nchi | osha |
| seri | simu | soko | twai | akili | amani |
| asili | barua | bendi | bloke | chaki | chuki |
| chuma | chupa | chupi | daima | fedha | funzi |
| gundi | hamsi | hatua | ijayo | imani | jansa |
| juuya | kamba | kamwe | kemia | kesho | kiatu |
| kifua | kofia | kubwa | kumba | kweli | mamba |
| mbuzi | mdudu | mekno | mfuko | miaka | mkasi |
| mkate | mkoba | mraba | msitu | mtawa | mungu |
| mvuke | mwezi | nanga | ndege | ndevu | ndizi |
| ndogo | ndugu | ngazi | ngono | ngozi | nopya |
| nyota | nzuri | ofisi | panya | petye | picha |
| pombe | punda | pwani | rangi | shule | siagi |
| tembo | tumbo | uadui | uhuru | ukame | uongo |
| usiku | uyoga | viatu | wimbo | wingi | wingu |
| adhabu | bafuni | bahari | baiski | bunifu | chombo |
| choori | dakika | daraja | dizeli | farasi | furaha |
| garisi | godoro | guruwe | hasira | hazini | ishara |
| ishiri | jeraha | jibini | jikoni | jokofu | jumatu |
| kaburi | kahawa | kalamu | katika | kelele | kichwa |
| kidole | kihozi | kijiko | kikapu | kimysa | kinywa |
| kisiwa | kitovu | kovuli | kuacha | kubale | kuhesa |
| kununa | kunywa | kuzama | mageho | maisha | mapafu |
| mashua | mbolea | mchawi | mchuzi | mlango | mpishi |
| mtirka | mwanga | mzungu | ndoora | nyange | nyeusi |
| nyundo | nyundu | rafiki | rombus | sabuni | sahani |
| samaki | sayari | sehemu | surali | tanuri | trekta |
| ufagio | ugomvi | ukweli | wakala | washia | welder |
| yatima | alizeti | baadaye | baharia | bandari | bilaska |
| bustani | chubani | filimbi | kartasi | kawaida | kengele |
| kitanda | kitande | kudhibi | kujenga | kukimba | kumbuka |
| kupanda | kusanya | kushoto | matumai | matumbo | mazishi |
| mchanga | mechezo | mgonjwa | msumari | mundamo | mvringo |
| mwanake | ndaniya | neyemba | punguza | shimoni | singizi |
| starehe | sufuria | sungura | takatak | tamasha | tumbili |
| uchorai | umasijo | wengine | zeituni | kuandika | kusikiza |
