# Supplementary material for: Failure to modulate reward prediction errors in declarative learning with theta (6 Hz) frequency transcranial alternating current stimulation
Source: PLoS One. 2020 Dec 3;15(12):e0237829. doi: 10.1371/journal.pone.0237829 (PMC7714179; doi:10.1371/journal.pone.0237829)
Supplement: S1 File — (DOCX) [file pone.0237829.s009.docx]

**S1 File. Sensations Questionnaire.**

| Did you experience any discomfort or annoyance during the electrical stimulation? Please answer the following questions regarding the different sensations and indicate the degree of intensity of your discomfort according to the following scale: | | | | | |
| --- | --- | --- | --- | --- | --- |
| • ***None* = I did not feel the described sensation (0)**  • ***Mild* = I mildly felt the described sensation (1)**  • ***Moderate* = I felt the described sensation (2)**  • ***Considerable* = I felt the described sensation to a considerable degree (3)**  • ***Strong* = I strongly felt the described sensation (4)** | | | | | |
|  | | | | | |
| Itching: | □ None | □ Mild | □ Moderate | □ Considerable | □ Strong |
| Pain: | □ None | □ Mild | □ Moderate | □ Considerable | □ Strong |
| Burning: | □ None | □ Mild | □ Moderate | □ Considerable | □ Strong |
| Warmth/Heat: | □ None | □ Mild | □ Moderate | □ Considerable | □ Strong |
| Pinching: | □ None | □ Mild | □ Moderate | □ Considerable | □ Strong |
| Metallic/Iron taste: | □ None | □ Mild | □ Moderate | □ Considerable | □ Strong |
| Fatigue: | □ None | □ Mild | □ Moderate | □ Considerable | □ Strong |
| Other_________________: | □ None | □ Mild | □ Moderate | □ Considerable | □ Strong |
| When did the discomfort begin? | | | | | |
| □ At the beginning of the block | | □ At approximately the middle of the block | | □ Towards the end of the block | |
| How long did it last? | | | | | |
| □ It stopped quickly | | □ It stopped in the middle of the block | | □ It stopped at the end of the block | |
| How much did these sensations affect your performance? | | | | | |
| □ Not at all | □ Slightly | □ Considerably | □ Much | □ Very much |  |
