# Supplementary material for: Failure to modulate reward prediction errors in declarative learning with theta (6 Hz) frequency transcranial alternating current stimulation
Source: PLoS One. 2020 Dec 3;15(12):e0237829. doi: 10.1371/journal.pone.0237829 (PMC7714179; doi:10.1371/journal.pone.0237829)
Supplement: Attachment — Submitted filename: ResponseToReviewers.docx [file pone.0237829.s011.docx]

**Response to Reviewers**

Dear Dr. Javadi,

We thank you and the five reviewers for the evaluation of our revised manuscript entitled “Failure to modulate reward prediction errors in declarative learning with theta (6 Hz) frequency transcranial alternating current stimulation”. We have carefully reviewed the comments and have revised the manuscript accordingly. Below you can find our answers to the reviewers’ comments to which we responded in a point-by-point fashion.

Sincerely,
Kate Ergo, Esther De Loof, Gillian Debra, Bernhard Pastötter, and Tom Verguts

**Reviewer 1**

**Comment:** Additionally, I appreciate that the distributional form of their data has been included in their figures, though I would recommend creating a clearer visual distinction in the data points in Fig. 2 panels (C) and (D), to separate incorrect vs. correct recognition (I believe they are current using triangle/circle markers, which are difficult to visually distinguish – the authors could consider jittering the vertical alignment to create more distinct columns of data points).

**Response:** We agree with Reviewer 1 that the triangle and circle markers were difficult to visually distinguish. Therefore, in the revised version of the manuscript, we have split up the Certainty panels of Figure 2 into four subfigures: certainty for correct recognitions in the real stimulation group, certainty for correct recognitions in the sham stimulation group, certainty for incorrect recognitions in the real stimulation group, and certainty for incorrect recognitions in the sham stimulation group. Each of these subfigures also contains individual data points representing mean certainty (gray circles for correct recognitions and gray triangles for incorrect recognitions) per condition for each participant.

**Comment:** As I mentioned in my original review, the authors certainly have the flexibility to present additional data that can clarify or support their results. For example, within-subject behavioral responses could be presented that demonstrate the extent to which certain subjects may or may not have used the full dynamic range of certainty ratings.

**Response:** We added the within-subject behavioral responses for the certainty ratings as Supporting Information (S3 Certainty Ratings for subjects 1 to 20, S4 Certainty Ratings for subjects 21 to 41, S5 Certainty Ratings for subjects 42 to 61, S6 Certainty Ratings for subjects 62 to 77).

**Comment:** Additionally, the authors could present a visualization that essentially captures the purpose of their LME, by showing the per-subject relationship between SRPE and certainty/accuracy. However, I would leave the need for such changes at the discretion of the editor.

**Response:** Because per-subject data can be noisy, we decided not to visualize the per-subject relationship between SRPE and Certainty/Accuracy.

**Reviewer 3**

**Comment:** The authors have addressed all my concerns with this revision. One comment, although not necessary to include in the manuscript, is that TMS will not always impact neural activity in a focal manner, as the effects of stimulation propagate throughout neural networks. The authors should consider this in potential future work (as described in the discussion).

**Response:** We agree with Reviewer 3. We now added to the Discussion section on page 20 that: “Even though the spatial resolution of TMS remains debated (Slotnick, 2013), it is more focal than tACS.”

**References**

Slotnick, S. (2013). *Controversies in cognitive neuroscience.* New York: Palgrave Macmillan.
